# Significant positive impact of duodenum‐preserving pancreatic head resection on the prevention of postoperative nonalcoholic fatty liver disease and acute cholangitis

**DOI:** 10.1002/ags3.12593

**Published:** 2022-06-30

**Authors:** Hiroyuki Kato, Yukio Asano, Masahiro Ito, Satoshi Arakawa, Masahiro Shimura, Daisuke Koike, Chihiro Hayashi, Kenshiro Kamio, Toki Kawai, Akihiko Horiguchi

**Affiliations:** ^1^ Department of Gastroenterological Surgery Fujita Health University School of Medicine Bantane Hospital Nagoya Japan

**Keywords:** duodenum‐preserving pancreatic head resection, FIB‐4 index, pancreaticoduodenectomy

## Abstract

**Aim:**

This study aimed to compare the incidence of postoperative nonalcoholic fatty liver disease (NAFLD), postoperative cholangitis, and fibrosis‐4 (FIB)‐4 index in patients who underwent duodenum‐preserving pancreatic head resection (DPPHR) and pancreaticoduodenectomy (PD) for low‐grade malignant tumors and verify the usefulness of DPPHR in preventing the occurrence of these disorders.

**Methods:**

This retrospective study included 70 patients who underwent PD (n = 39) and DPPHR (n = 31) between 2006 and 2018 for benign or low‐grade malignant tumors. The present study compared the preoperative background, cumulative incidence of postoperative NAFLD and cholangitis, and other biochemical markers, including the FIB‐4 index. Subanalysis by propensity score matching (PSM) analysis was conducted to minimize treatment selection bias.

**Results:**

In terms of the cumulative incidence of NAFLD, the 5‐y incidence was significantly lower in the DPPHR group than in the PD group both before (10% vs 38%, *P* = .002) and after (13% vs 38%, *P* = .008) matching. Multivariate analyses identified DPPHR as the only independent preventive factor for postoperative NAFLD (hazard ratio: 0.160, 95% confidence intervals: 0.034–0.76, *P* = .021). The 5‐y cumulative incidence of postoperative cholangitis was significantly higher in the PD group than in the DPPHR group before (51% vs 3%, *P* < .001) and after (49% vs 4%, *P* < .001) matching. The FIB‐4 index at 12 mo postoperatively was significantly better in the DPPHR group than in the PD group (1.45 vs 2.35, *P* = .006) before matching.

**Conclusion:**

Preservation of the duodenum and bile duct may contribute to preventing long‐term postoperative NAFLD and cholangitis, and liver fibrosis for benign or low‐grade malignant pancreatic head tumors.

## Graphical abstract

This study aimed to compare the incidence of postoperative NAFLD, postoperative cholangitis, and FIB‐4 index in patients treated with DPPHR and PD for low‐grade malignancy and prove the usefulness of DPPHR in preventing the development of these disorders.

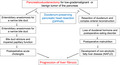

## 1 INTRODUCTION

With the development of surgical techniques in recent y, short‐term complications and mortality after pancreaticoduodenectomy (PD) have decreased.^1^, ^2^ Conversely, long‐term complications, such as postoperative malnutrition, resulting in fatty liver disease and postoperative cholangitis, greatly reduce the quality of life (QOL) of patients with low‐grade malignant tumors whose prognosis is favorable and whose long‐term survival is anticipated. Recently, laparoscopic surgical techniques have improved, and laparoscopic PD and robotic PD have been widely performed for low‐grade malignant pancreatic tumors.^3^, ^4^, ^5^ Even if these advancements allow PD to be performed with smaller wounds and less invasiveness, the incidence of complications, such as fatty liver disease and postoperative cholangitis, is not theoretically reduced. In addition, low‐grade malignant tumors, such as solid pseudopapillary neoplasm (SPN) and pancreatic neuroendocrine neoplasm (NEN), are expected to have long‐term survival in most cases and are often detected at a young age, making it a dilemma for surgeons as to whether PD is indeed necessary and can be indicated. This is because the pancreatic head is anatomically adjacent to the bile duct and duodenum, and therefore resected to conveniently remove the tumor.

Postoperative nonalcoholic fatty liver disease (NAFLD) develops in 30%–40% of patients after PD.^6^ Although it is usually a benign disease and has been mostly ignored, recently there have been reports of cases of long‐term progression to nonalcoholic steatohepatitis (NASH), leading to cirrhosis and death,^7^, ^8^ which is a problem that must be solved. Pancreatic exocrine function replacement is believed to be an effective treatment for NAFLD/NASH, but its effect remains controversial.^9^

Recently, the usefulness of the fibrosis (FIB)‐4 index as an indicator of liver fibrosis in patients with NAFLD has been widely reported,^10^, ^11^ and its value is strongly correlated with pathological liver fibrosis. Therefore, the clinical relevance of the FIB‐4 index as an indicator of liver fibrosis in patients with NAFLD after pancreatectomy remains unclear.

The development of postoperative acute cholangitis after PD may be caused by hepaticojejunostomy, and one possible cause is the reflex of intestinal fluid and intestinal gas into the intrahepatic bile duct, resulting in increased intraductal pressure and bacteremia.^12^ Anastomotic stenosis is also a major cause of cholangitis, and patients with a preoperative narrow bile duct diameter in benign disease are at high risk of recurrent cholangitis.^13^ Therefore, patients with almost no jaundice, no bile duct dilatation, and low‐grade tumors, such as primitive neuroectodermal tumor, solid‐pseudopapillary neoplasm, or intraductal papillary mucinous neoplasm, are likely to have a high risk of postoperative cholangitis, and their postoperative QOL might be greatly impaired. Moreover, postoperative cholangitis is recognized as the second‐most common cause of NAFLD in PD, which might trigger the transition to NASH from NAFLD.^8^

Duodenum‐preserving pancreatic head resection (DPPHR) was first described by Beger et al^14^ as a procedure for severe chronic pancreatitis, and its indications were expanded to include pancreatic head tumors. However, DPPHR requires careful preservation of the pancreatic head arterial arcades and is not widely used compared with PD because of the difficulty of its indication, especially when the tumor is close to the intrapancreatic bile duct or arterial arcades. Therefore, there are very few studies on the postoperative nutritional status and complications after DPPHR and no reports comparing the frequency of postoperative NAFLD and postoperative cholangitis with those of PD. We previously reported that exocrine pancreatic function, that is, fat absorption function, was significantly preserved in patients who underwent DPPHR compared with those who underwent PD.^15^ Therefore, we hypothesized that patients who underwent DPPHR would have significantly less postoperative hepatic damage caused by NAFLD or cholangitis, even though the pancreatic head was resected as much as in PD.

This study aimed to compare the incidence of postoperative NAFLD, postoperative cholangitis, and the FIB‐4 index in patients treated with DPPHR and PD for low‐grade malignancy and prove the usefulness of DPPHR in preventing the development of these disorders. To the best of our knowledge, this is the first study to prove the importance of preservation of the bile duct and duodenum to prevent postoperative NAFLD, acute cholangitis, and liver fibrosis.

## 2 METHODS

Between 2006 and 2018, 75 patients with a diagnoses of benign or low‐grade malignant tumors underwent pancreatic head resections (PD in 41 and DPPHR in 34). However, in five patients (two for PD, three for DPPHR), clinical data were missing, since the postoperative follow‐ups had been performed at other hospitals after discharge. This retrospective study included 70 patients who underwent PD (n = 39) and DPPHR (n = 31).

The Medical Ethics Committee of Fujita Health University School of Medicine approved the study protocol (HM17‐372). The median computed tomography (CT) attenuation values of the liver parenchyma were measured using a 5‐point scale of certainty on plain CT preoperatively and at 12 mo postoperatively. We defined NAFLD as a liver parenchyma CT value <50 Hounsfield units (HU), according to previous reports.^16^, ^17^, ^18^ The FIB‐4 index was calculated using the following formula: age (y) × AST (U/L)/[platelet (1000/μL) × √ALT(U/L)].^19^ Although the FIB‐4 index represents liver fibrosis, it was not used to define NAFLD in this study. Postoperative cholangitis is defined as cholangitis categorized as a suspected or definite diagnosis according to the Tokyo Guideline 2018,^20^ which required hospitalization and antibiotic treatment. In the present study we compared the preoperative background, incidence of postoperative NAFLD, cumulative incidence of postoperative cholangitis, and other biochemical markers, including the FIB‐4 index.

As for the surgical indication for DPPHR, we can adapt DPPHR as long as the arterial arcade and bile duct are considered preservable. However, DPPHR often cannot be indicated if the tumor is either adjacent to the bile duct or is too large to preserve the arterial arcade in the pancreatic head, regardless of the degree of its malignancy. If a lymph node dissection is considered necessary due to swelling of the peripancreatic lymph node, DPPHR is not indicated, and PD remains the only option.

**FIGURE 1:**
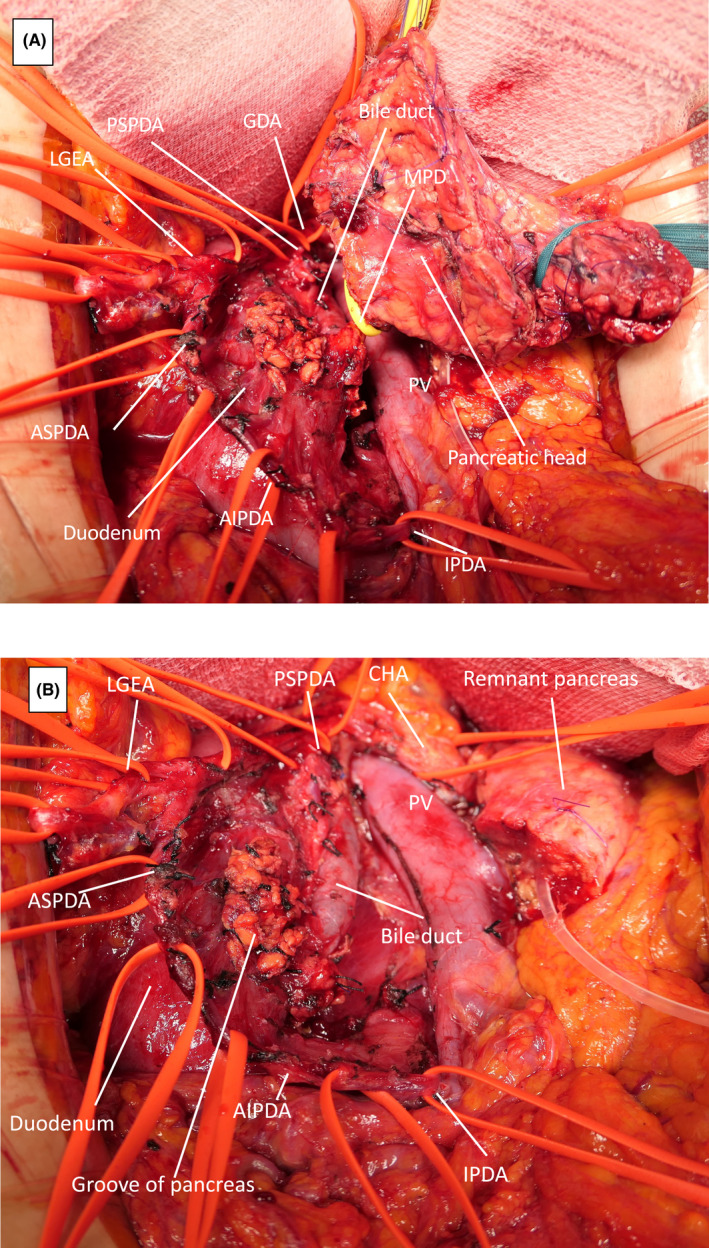
Intraoperative findings of DPPHR. (A) Just before resection of the pancreatic head. (B) After resection. Abbreviations: AIPDA, anterior IPDA; ASPDA, anterior superior pancreaticoduodenal artery; BD, bile duct; CHA, common hepatic artery; DPPHR, duodenum‐preserving pancreatic head resection; GDA, gastroduodenal artery; IPDA, inferior pancreaticoduodenal artery; LGEA, left gastroepiploic artery; MPD, main pancreatic duct; PIPDA, posterior IPDA; PSPDA, posterior superior pancreaticoduodenal artery; PV, portal vein

Regarding the surgical procedure of DPPHR, a precise surgical technique was described in a previous study.^15^ After laparotomy, both the gastric and duodenocolic ligaments were dissected without Kocher's maneuver to preserve the venous drainage from the duodenum. Thereafter, the common hepatic artery and gastroduodenal artery were exposed. The pancreas was divided above the portal vein, and a polyvinyl tube was inserted into the main pancreatic duct (MPD) of the remnant pancreas. During resection of the uncinate portion, the inferior pancreaticoduodenal artery (IPDA) was taped, and the pancreatic branches of the anterior inferior pancreaticoduodenal artery (AIPDA) were ligated and divided toward the papilla of Vater, preserving the branches of the AIPDA to the duodenum. While ligating and dividing the pancreatic branches of the ASPDA toward the papilla of Vater, the duodenal branches were preserved. After taping the bile duct at the upper margin of the pancreas, the pancreatic head was dissected at the anterior wall of the bile duct toward the papilla of Vater, and the confluence of the MPD was exposed. The MPD was ligated, and the pancreatic head was removed with preservation of the bile duct and duodenum **(**Figure 1 **)**. Reconstruction was performed by end‐to‐side pancreaticojejunostomy using a Rouxen‐Y jejunal loop. For PD reconstruction, the modified Child method with Braun anastomosis was used. For pancreatojejunostomy in both procedures, first‐layer anastomosis was performed through duct‐to‐mucosa anastomosis with 6–8 interrupted sutures using 5–0 PDS II (Ethicon, Johnson & Johnson, Raritan, NJ, USA). Second‐layer anastomosis was performed using the modified Kakita procedure with six sutures using 3–0 Prolene.^21^ A 5‐F external pancreatic stent tube was inserted into the remnant main pancreatic duct.

All statistical analyses were conducted using SPSS for Macintosh (v. 24.0; IBM, Armonk, NY, USA). Continuous variables were expressed as medians and ranges. The statistical significance of the continuous variables was determined using the Mann–Whitney *U* test. Categorical variables were compared using Pearson's chi‐squared test. The risk factors associated with NAFLD were analyzed using univariate and multivariate (logistic regression) analyses. Only variables with a *P*‐value <.1 on univariate analysis were included in the multivariate analysis. The overall cumulative incidence of postoperative NAFLD and acute cholangitis were calculated using the Kaplan–Meier method, and the Kaplan–Meier curves were compared using the log‐rank test. To minimize treatment selection bias, propensity score matching subanalysis was used. Propensity scores were developed by logistic regression analysis using the preoperative patient age. This was used because it was the only preoperative factor in which univariate analysis showed a statistically significant difference between the PD and DPPHR groups. The PD and DPPHR patients were then paired 1:1 on these propensity scores using exact matching. A standard caliper size of 0.2 × standard deviation of the propensity score was used.

## 3 RESULT

**FIGURE 2:**
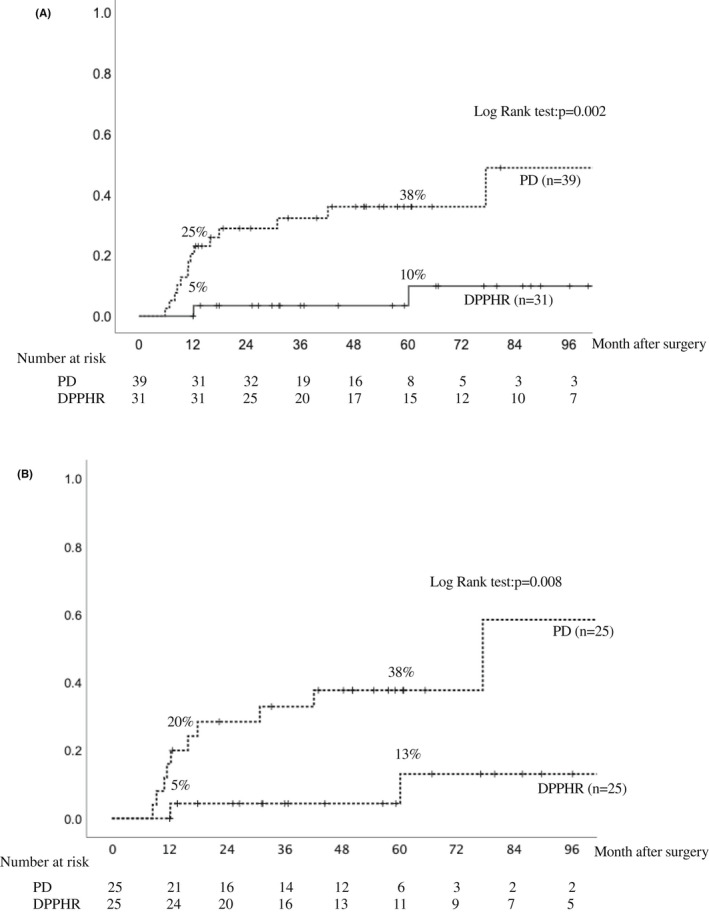
Comparison of cumulative incidence of NAFLD by surgical procedures. (A) Before propensity score matching. (B) After matching. Abbreviations: DPPHR, duodenum‐preserving pancreatic head resection; NAFLD, nonalcoholic fatty liver disease; PD, pancreaticoduodenectomy

Table 1 shows a comparison of perioperative characteristics between the DPPHR and PD groups. The patients who underwent DPPHR were significantly younger than those who underwent PD (57.5 vs 68.2; *P* = .003). Otherwise, there were no significant differences in terms of sex, primary disease, preoperative FIB‐4 index, operative time, and blood loss. In Table 2 we compare the backgrounds of the two groups after adjusting for age using propensity score matching. All perioperative backgrounds, including age, were comparable after matching. As shown in Table 3, the CT attenuation value at 12 mo postoperatively was significantly higher in the DPPHR group than that in the PD group (60.0 vs 53.1; *P* = .005), and the FIB‐4 index at 12 mo postoperatively was significantly better in the DPPHR group than that in the PD group (1.45 vs 2.35; *P* = .006) before matching. Concurrently, the serum albumin level was also significantly better in the DPPHR group than in the PD group (4.2 vs 3.9; *P* = .003), and the prognostic nutrition index tended to be better in the DPPHR group, although the difference was not significant. However, these statistically significant differences disappeared in the albumin value and the FIB‐4 index after matching, and only the CT attenuation value remained as a significant factor after matching. When the cumulative incidence of NAFLD was compared between the two groups, the 5‐y cumulative incidence was significantly lower in the DPPHR group than in the PD group (10% vs 38%; *P* = .002) (Figure 2A). Even after propensity score matching, the cumulative incidence was significantly lower in the DPPHR group than in the PD group (13% vs 38%; *P* = .008) (Figure 2B). Moreover, based on the results of univariate and multivariate analyses to identify the perioperative preventive factor of NAFLD in the cohort after matching, DPPHR was identified as the only independent preventive factor for postoperative NAFLD (hazard ratio: 0.160; *P* = .021) (Table 4).

**TABLE 1:** Comparison of patient backgrounds between the DPPHR and PD groups

	DPPHR (n = 31)	PD (n = 39)	*P*‐value
Preoperative variables			
Age (y)	57.5 ± 15.8	68.2 ± 10.8	.003
Gender (male/female)	17/15	25/14	.432
Body mass index	21.9 ± 3.4	20.5 ± 2.9	.112
Diagnosis (IPMN/NEN/SPN/SCN/others)	23/5/3/0/0	34/3/0/1/1	.147
CT attenuation value (HU)	58.6 ± 5.6	59.9 ± 9.5	.367
Albumin (g/dL)	4.3 ± 0.33	4.1 ± 0.55	.238
Total lymphocyte count (/mm^3^)	1644 ± 661	1543 ± 618	.533
Hemoglobin (g/dL)	13.8 ± 1.4	13.1 ± 1.4	.072
Prognostic nutrition index	48.8 ± 10.0	47.9 ± 6.1	.148
Aspartate aminotransferase (AST) (IU/L)	21.8 ± 9.6	23.3 ± 6.9	.207
Alanine transaminase (ALT) (IU/L)	18.2 ± 8.6	20.4 ± 9.2	.225
Platelet count (10^3^/μL)	211 ± 62	243 ± 11	.083
FIB‐4 index	1.54 ± 0.73	1.67 ± 0.82	.527
Intraoperative variables			
Operative time (min)	455 ± 89	469 ± 206	.525
Blood loss (g)	358 ± 306	454 ± 791	.661
Postoperative variables			
Clinically relevant pancreatic fistula (%)	16.1% (5/31)	20.5% (8/39)	.639
Complication with Clavien–Dindo grade ≥3 (%)	29% (9/31)	28% (11/39)	.939
Postoperative exocrine enzyme supplementation (yes/no)	31/0	37/2	.578

Abbreviations: DPPHR, duodenum‐preserving pancreatic head resection; Fib‐4, Fibrosis‐4; HU, Hounsfield Unit; IPMN, intraductal papillary mucinous neoplasm; NEN, neuroendocrine neoplasm; PD, pancreaticoduodenectomy; SCN, serous cystic neoplasm; SPN, solid pseudopapillary neoplasm.

**TABLE 2:** Comparison of patient backgrounds between the DPPHR and PD groups after propensity score matching

	DPPHR (n = 25)	PD (n = 25)	*P*‐value
Preoperative variables			
Age (y)	62.8 ± 11.3	64.2 ± 10.6	.698
Gender (male/female)	15/10	16/9	.771
Body mass index	21.0 ± 3.0	23.1 ± 3.3	.064
Diagnosis (IPMN/NEN/SPN/SCN/others)	20/4/1/0/0	21/2/0/1/1	.151
CT attenuation value (HU)	57.8 ± 5.7	61.8 ± 7.6	.128
Albumin	4.2 ± 0.31	4.3 ± 0.4	.651
Total lymphocyte count	1524 ± 532	1675 ± 653	.378
Hemoglobin	13.7 ± 1.5	13.4 ± 1.3	.289
Prognostic nutrition index	47.5 ± 10.7	49.9 ± 4.8	.698
Aspartate aminotransferase (AST) (IU/L)	23.4 ± 10.7	21.4 ± 6.4	.425
Alanine transaminase (ALT) (IU/L)	20.0 ± 8.7	19.3 ± 6.9	.992
Platelet count (10^3^/μL)	207 ± 66	234 ± 122	.248
FIB‐4 index	1.72 ± 0.68	1.63 ± 0.98	.327
Intraoperative variables			
Operative time (min)	447 ± 80	491 ± 237	.788
Blood loss (g)	375 ± 323	327 ± 264	.549
Postoperative variables			
Clinically relevant pancreatic fistula (%)	16.0% (4/25)	28.0% (7/25)	.306
Complication with Clavien–Dindo grade ≥3 (%)	28% (7/25)	40% (10/25)	.370
Postoperative exocrine enzyme supplementation (yes/no)	25/0	23/2	.478

Abbreviations: DPPHR, duodenum‐preserving pancreatic head resection; Fib‐4:Fibrosis‐4; HU, Hounsfield Unit; IPMN, intraductal papillary mucinous neoplasm; NEN, neuroendocrine neoplasm; PD, pancreaticoduodenectomy; SCN, serous cystic neoplasm; SPN, solid pseudopapillary neoplasm.

**TABLE 3:** Comparison of postoperative variables between the DPPHR and PD groups before and after propensity score matching

	Before matching			After matching		
	DPPHR (n = 31)	PD (n = 39)	*P*‐value	DPPHR (n = 25)	PD (n = 25)	*P*‐value
Postoperative variables						
CT attenuation value (HU)	60.0 ± 4.5	53.1 ± 12.2	.005	60.0 ± 4.8	53.1 ± 12.4	.010
Albumin (g/dL)	4.2 ± 0.28	3.9 ± 0.54	.003	4.2 ± 0.30	4.1 ± 0.36	.105
Total lymphocyte count (/mm^3^)	1484 ± 495	1574 ± 656	.593	1492 ± 454	1696 ± 674	.159
Hemoglobin (g/dL)	12.9 ± 1.3	12.0 ± 1.9	.083	13.0 ± 1.3	12.4 ± 1.6	.312
Prognostic nutrition index	48.8 ± 10.0	47.9 ± 6.1	.073	47.2 ± 11.1	48.4 ± 4.9	.803
Aspartate aminotransferase (AST) (IU/L)	28.8 ± 21.0	39.4 ± 46.8	.207	31.3 ± 23.1	42.6 ± 55.9	.788
Alanine transaminase (ALT) (IU/L)	29.3 ± 19.5	30.3 ± 25.9	.225	32.2 ± 20.9	30.9 ± 27.4	.326
Platelet count (10^3^/μL)	225 ± 67	230 ± 79	.083	226 ± 73	229 ± 86	.918
FIB‐4 index	1.45 ± 0.73	2.35 ± 2.23	.006	1.61 ± 0.69	2.34 ± 2.50	.219

Abbreviations: DPPHR, duodenum‐preserving pancreatic head resection; Fib‐4, Fibrosis‐4; HU, Hounsfield Unit; NAFLD, nonalcoholic fatty liver disease; PD, pancreaticoduodenectomy.

**TABLE 4:** Uni‐ and multivariate analyses for identifying the influential factor of postoperative NAFLD after propensity score matching

	Univariate			Multivariate		
	Hazard ratio	95% CI	*P*‐value	Hazard ratio	95% CI	*P*‐value
Preoperative variables						
Age (y)	1.021	0.963–1.082	.493			
Sex (male)	1.983	0.632–6.222	.241			
Diagnosis (IPMN)	1.210	0.264–5.557	.806			
Preoperative body mass index	1.140	0.942–1.380	.177			
CT attenuation value (HU)	1.019	0.939–1.106	.650			
Albumin	1.382	0.277–6.904	.693			
Total lymphocyte count	1.001	1.000–1.002	.074			
Hemoglobin	1.009	0.661–1.541	.967			
Prognostic nutrition index	1.058	0.946–1.183	.321			
Aspartate aminotransferase (IU/L)	0.998	0.940–1.060	.949			
Alanine transaminase (IU/L)	0.987	0.917–1.063	.736			
Platelet count (10^3^/μL)	0.994	0.923–1.070	.864			
FIb‐4 index	1.414	0.767–2.607	.267			
Intraoperative variables						
Surgical procedures (DPPHR)	0.160	0.0350–0.739	.019	0.160	0.0341–0.763	.021
Operative time (min)	0.999	0.995–1.003	.683			
Blood loss (g)	0.999	0.996–1.002	.449			
Postoperative variables						
Clinically relevant pancreatic fistula (yes)	1.729	0.517–5.781	.374			
Complication with Clavien–Dindo grade ≥3	3.051	0.965–9.651	.058	2.959	0.915–9.615	.070
Postoperative exocrine enzyme supplementation (yes)	0.458	0.058–3.586	.457			

Abbreviations: CI, confidence interval; DPPHR, duodenum‐preserving pancreatic head resection; Fib‐4, Fibrosis‐4; HU, Hounsfield Unit; NAFLD, nonalcoholic fatty liver disease; PD, pancreaticoduodenectomy.

**FIGURE 3:**
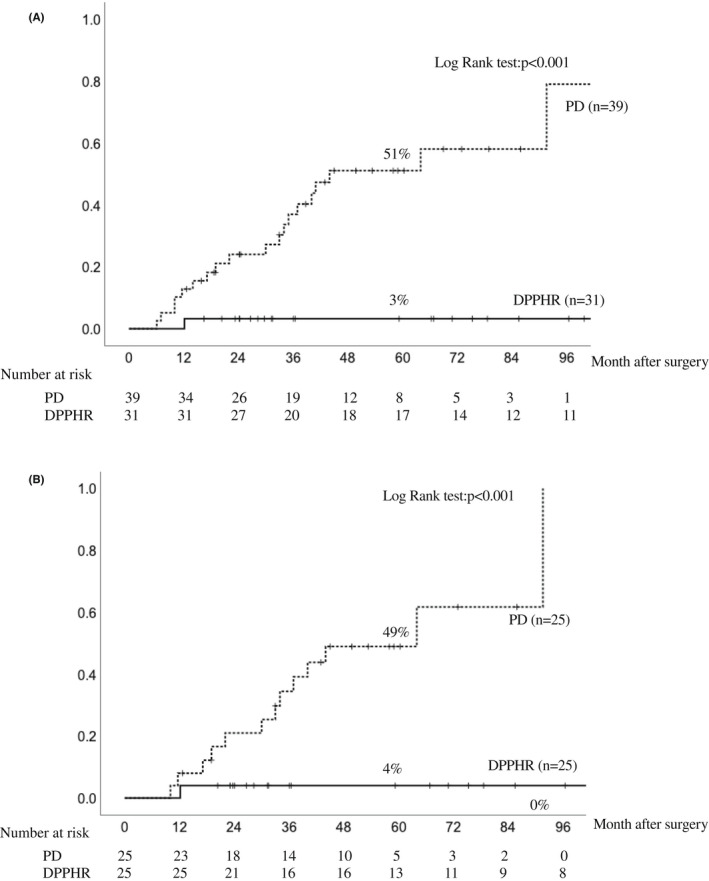
Comparison of cumulative incidence of postoperative cholangitis by surgical procedures. (A) Before propensity score matching. (B) After matching. Abbreviations: DPPHR: duodenum‐preserving pancreatic head resection; PD, pancreaticoduodenectomy

The 5‐y cumulative incidence of postoperative cholangitis was significantly higher in the PD group than in the DPPHR group before (51% vs 3%; *P* < .001) and after (49% vs 4%; *P* < .001) propensity score matching (Figure 3). The mean follow‐up period of total cases was 61.2 mo. In the DPPHR group, postoperative cholangitis developed in only one of 31 patients. Of the 19 patients who developed postoperative cholangitis after PD, four developed refractory cholangitis with recurrence of more than three times, and four patients had a positive blood culture due to cholangitis. One patient developed hepatolithiasis.

In terms of local recurrence of tumors, for reference there were no obvious recurrent cases in 70 cases; however, three patients (one in the DPPHR group and two in the PD group) whose primary tumor was an intraductal papillary mucinous adenoma developed remnant pancreatic cancer.

## 4 DISCUSSION

In this study the following new insights were obtained: (a) preservation of the duodenum and bile duct by DPPHR significantly reduced the incidence of postoperative NAFLD, and DPPHR was selected as an independent preventive factor of NAFLD. (b) The cumulative incidence of postoperative cholangitis was extremely low in the DPPHR group, while there were cases of recurrent cholangitis and sepsis over a long period after surgery in the PD group. (c) The FIB‐4 index was significantly lower in the DPPHR group at 12 mo postoperatively in the cohort before propensity score matching.

NAFLD is caused by visceral obesity^22^ and lifestyle‐related diseases^23^ due to chronic overnutrition, and its incidence is increasing annually. Some cases of NAFLD lead to inflammation and progressive liver fibrosis, resulting in NASH. The concept of NASH was proposed by Ludwig et al^24^in 1980, and it is a disease with pathological findings similar to those of alcoholic hepatitis, which progressively leads to cirrhosis and liver failure in nondrinkers. NAFLD that develops after PD differs from ordinary NAFLD in terms of cause, and its major characteristic is that it develops without insulin resistance due to worsening nutritional status postoperatively. NAFLD after PD is thought to be caused by malnutrition induced by impaired fat absorption due to pancreatic exocrine insufficiency or deficiency of duodenal hormones or an eating disorder due to complicated gastrointestinal reconstruction, leading to increased conversion of carbohydrates to fats in the liver, similar to that in fatty liver disease caused by starvation, such as kwashiorkor.^6^ Regarding the treatment for NAFLD after PD, pancreatic exocrine enzyme replacement is considered the most promising treatment, with a recent randomized control study showing that it also significantly prevents NAFLD after PD.^25^ Therefore, pancreatic exocrine enzyme replacement therapy is routinely administered after a pancreatectomy at our institution.

Given that the cause of NAFLD is malnutrition, the lower incidence of NAFLD after DPPHR could be explained by the preservation of the duodenal and bile duct in the DPPHR group, thus preserving postoperative nutrition by maintaining normal gastroduodenal function and physiological bile secretion. Lue et al^26^ revealed that the incidence of NAFLD is significantly lower in DP than in PD, even though the amount of resection of the pancreatic parenchyma is greater in DP than in PD. The absence of gastrointestinal reconstruction and preservation of the duodenum in DP may be essential for the low incidence of NAFLD. Beger et al^27^ recently compared the incidence of postoperative pancreatic exocrine insufficiency and new‐onset diabetes mellitus in the DPPHR and PD groups using a systematic review approach and reported significantly lower rates in the DPPHR group, which supports our hypothesis. Furthermore, they reported that the postoperative secretion of cholecystokinin, which is secreted from the duodenum and stimulates the secretion of pancreatic and bile juices, was significantly higher in the DPPHR group than that in the PD group. Although these articles may indirectly support our hypothesis, we considered the comparison of the incidence of NAFLD between DPPHR and PD to be crucial to clinically prove this hypothesis. To the best of our knowledge, this is the first study to show that preservation of the duodenum and bile duct plays a significant protective role in the development of NAFLD.

Postoperative cholangitis frequently and significantly impairs the patient's QOL. In the present study, the cumulative incidence of postoperative cholangitis that required hospitalization in the PD group was >50% during the follow‐up period. This may be due to the fact that the primary diseases of enrolled patients were benign or low‐grade malignant tumors without jaundice or bile duct dilation due to tumor obstruction preoperatively; thus, the incidence of cholangitis may have been higher since hepaticojejunostomy of the skinny bile ducts led to the development of strictures over a long period. Previous studies report that a small the bile duct diameter is the most important preoperative risk factor for a biliary stricture causing cholangitis after PD.^28^, ^29^ Therefore, if ampullary function could be preserved by performing DPPHR, as in the results of this study, patients will not have to be anxious of the risk of postoperative cholangitis, resulting in more favorable QOL, especially for young patients with a long‐term prognosis. Regarding the incidence of postoperative cholangitis after DPPHR, Umemoto et al^30^ also reported that cholangitis did not develop in 12 patients who underwent DPPHR, which is consistent with the results obtained in our study.

The role of postoperative cholangitis in the development of fatty liver disease and transition from NAFLD to NASH remains controversial. However, it has been assumed that the influx of enteric bacteria into the intrahepatic bile ducts activates Kupffer cells in the liver and promotes the uptake of fat droplets into hepatocytes.^31^, ^32^ Bacterial translocation due to cholangitis or bacterial enteritis is also considered one of the factors of the second hit that leads to NASH.^33^ Whether postoperative cholangitis is associated with the development of postpancreatectomy NAFLD requires further investigation.

The FIB‐4 index is a scoring system that combines blood test data to evaluate the degree of liver fibrosis.^34^, ^35^ In early detection and follow‐up, it is important to evaluate the degree of progression of liver fibrosis, which is considered to have the strongest correlation to life prognosis, rather than the degree of fatty liver disease. Liver biopsy is essential for the definitive diagnosis of NASH, which has the risk of progression to cirrhosis or liver cancer; however, it is not practical to perform liver biopsy in all patients with NAFLD. In addition, liver biopsy is associated with hospitalization and sampling error; therefore, the FIB‐4 index is a very useful tool to more easily assess the degree of liver fibrosis. In fact, the results of this study showed that the FIB4‐index at 12 mo postoperatively was significantly lower in the DPPHR group, indicating that preservation of the duodenum and bile ducts contributes to the suppression of liver fibrosis. However, this significant difference disappeared in the cohort after propensity score matching. The reasons for this may be associated with the fact that age was included in the formula for calculating the FIB‐4 index. As the age between the two groups was adjusted to be equal and the number of cases decreased from 70 to 50 cases by matching, the statistical significance was abolished. Therefore, further case accumulation is needed to verify whether DPPHR reduces the degree of liver fibrosis when compared with PD.

This study had several limitations. First, this was a single‐center retrospective study; therefore, it might be considered only as an exploratory investigation. Therefore, a large‐scale multicenter study is desirable to demonstrate the reproducibility of the results of this study. Second, DPPHR is difficult to perform in patients with tumors close to the bile duct or in cases where the pancreatic arterial arcades cannot be preserved, and it is not indicated for all lesions located in the pancreatic head. Depending on the size and location of the tumor, PD may have to be performed in several cases. Nonetheless, based on the results of this study we believe that patients will obtain a large benefit in the long term if DPPHR is performed rather than PD.

In conclusion, preservation of the duodenum and bile duct may contribute to preventing the development of NAFLD for up to 1 y postoperatively and long‐term postoperative cholangitis for benign or low‐grade pancreatic head tumors. As there were no cases of recurrence caused by the procedures, DPPHR should be considered for these tumors whenever possible.

## DISCLOSURE

Funding: The present study was not funded by any organization.

Conflict of Interest: Hiroyuki Kato and Akihiko Horiguchi are Editorial Board members.

Author Contributions: HK analyzed and drafted the article. UA and AH participated in the data collection and assisted with data interpretation. MI, SA, MS, DK, CH, KK, TK, and AH reviewed and revised the article. All authors read and approved the final article.

Ethics: The Medical Ethics Committee of Fujita Health University School of Medicine approved the study protocol (HM17‐372).

Informed Consent: informed consent in this study was obtained through an opt‐out method.
